# Histogram Analysis of Perfusion Parameters from Dynamic Contrast-Enhanced MR Imaging with Tumor Characteristics and Therapeutic Response in Locally Advanced Rectal Cancer

**DOI:** 10.1155/2018/3724393

**Published:** 2018-08-13

**Authors:** Dong Myung Yeo, Soon Nam Oh, Moon Hyung Choi, Sung Hak Lee, Myung Ah Lee, Seung Eun Jung

**Affiliations:** ^1^Department of Radiology, Daejeon St. Mary's Hospital, The Catholic University of Korea, Republic of Korea; ^2^Departments of Radiology, Seoul St. Mary's Hospital, The Catholic University of Korea, Republic of Korea; ^3^Hospital Pathology, Seoul St. Mary's Hospital, The Catholic University of Korea, Republic of Korea; ^4^Division of Oncology, Department of Internal Medicine, Seoul St. Mary's Hospital, The Catholic University of Korea, Republic of Korea

## Abstract

**Purpose:**

To explore the role of histogram analysis of perfusion parameters from dynamic contrast-enhanced magnetic resonance imaging (DCE-MRI) based on entire tumor volume in discriminating tumor characteristics and predicting therapeutic response in rectal cancer.

**Materials and Methods:**

Thirty-seven DCE-MRIs of locally advanced rectal cancer patients who received chemoradiation therapy (CRT) before surgery were analyzed by pharmacokinetic model for quantification and histogram analysis of perfusion parameters. The results were correlated with tumor characteristics including EGFR expression, KRAS mutation, and CRT response based on the pathologic tumor regression grade (TRG).

**Results:**

The area under the contrast agent concentration-time curve (AUC) skewness was significantly lower in patients with node metastasis. The v_p_ histogram parameters were significantly higher in group with perineural invasion (PNI). The receiver operating characteristics (ROC) curve analyses showed that mode v_p_ revealed the best diagnostic performance of PNI. The values of K^trans^ and k_ep_ were significantly higher in the group with KRAS mutation. ROC curve analyses showed that mean and mode K^trans^ demonstrated excellent diagnostic performance of KRAS mutation. DCE-MRI parameters did not demonstrate statistical significance in correlating with TRG.

**Conclusion:**

These preliminary results suggest that a larger proportion of higher AUC skewness was present in LN metastasis group and a higher v_p_ histogram value was present in rectal cancer with PNI. In addition, K^trans^ and k_ep_ histogram parameters showed difference according to the KRAS mutation, demonstrating the utility of the histogram of perfusion parameters derived from DCE-MRI as potential imaging biomarkers of tumor characteristics and genetic features.

## 1. Introduction

Perfusion parameters from dynamic contrast-enhanced magnetic resonance imaging (DCE-MRI) based on pharmacokinetic modeling have been investigated as promising imaging biomarkers to assess tumor biologic properties and behaviors and to monitor and predict therapeutic effects on the basis of tumor perfusion. Among them, the widely used perfusion parameters extracted from the two-compartment pharmacokinetic Tofts model [1] have K^trans^ [volume transfer constant between blood plasma and the extravascular extracellular space (EES), which is determined by blood flow and vascular permeability], k_ep_ (rate constant or reflux rate between blood plasma and EES, k_ep_=K^trans^/v_e_), v_e_ (fractional EES volume), v_p_ (fractional plasma volume), and area under the contrast agent concentration-time curve (AUC, total amount of contrast agent).

In rectal cancer, change in K^trans^ after neoadjuvant chemoradiation therapy (CRT) in locally advanced rectal cancer has been correlated with pathologically favorable responses in previous studies [2, 3]. In addition, the initial K^trans^ measured by preoperative DCE-MRI was also reported to be a useful marker in predicting good response to neoadjuvant CRT [2, 4].However, contradictory findings have also been reported. Kim et al. [3] found no significant difference in the initial value or change in perfusion parameters between good responders and nonresponders of CRT or between pathologic complete responders and noncomplete responders. Furthermore, correlations of TNM stage with perfusion parameters also showed discrepant results[5, 6]. Based on these previous studies, there are many factors that influence the variable results of tumor perfusion analysis using DCE-MRI such as intrinsic limits in a simplified pharmacokinetic model, measurement error of arterial input function, difference among postprocessing software, small number of cases, sampling bias of region of interest (ROI), or inherent tumor heterogeneity [7, 8].In order to reduce and avoid sampling bias and to overcome limited results arising from intrinsic tumor heterogeneity, entire lesion-ROI analysis has been demonstrated to be more a reproducible method with low interobserver variability [8, 9]. Furthermore, histogram analysis of the entire tumor can provide direct information on the heterogeneity of the tumor using the value of each pixel or voxel. In recent studies, histogram analysis based on MRI has been performed in various areas of cancer research [10–13].To our knowledge, volume-based histogram analysis of perfusion maps in rectal cancer has not been well demonstrated in the literature. The purpose of our study was to explore the role of histogram analysis of DCE-MRI based on entire tumor volume in discriminating tumor characteristics and predicting neoadjuvant CRT response.

## 2. Materials and Methods

### 2.1. Patient Population

The institutional review board approved this retrospective study, and patient informed consent was waived. From December 2011 to March 2015, 167 consecutive patients with locally advanced rectal cancer (stages II (cT3-4, N0, M0) and III (cT1-4, N+, M0) were treated with CRT at our institution. The inclusion criteria for our study were biopsy-proven adenocarcinoma of the rectum treated with neoadjuvant CRT followed by resection of the tumor, adequate MR examinations to delineate the rectal cancer that included sequences to obtain a perfusion map before CRT, and availability of detailed surgical and histopathologic reports. In total, 37 met these inclusion criteria and formed the population of this study. There were 25 men and 12 women. The median age was 61 years (range, 29-84 years).The other 130 patients were excluded for no obtainment of MR sequences for perfusion map (n = 96), image distortion by motion or metallic artifact (n = 21), and inadequate histopathologic reports (n = 13). Preoperative MR imaging including sequences to produce perfusion map was not performed for the following reasons: other MR equipment which was not available to produce perfusion map was used (n = 74), and patients were not expected to be treated neoadjuvant CRT after understaging by computed tomography and colonoscopy (n = 22).

Among this cohort, one patient was reported elsewhere; it was addressed whether only mean values of quantitative parameters derived from DCE-MRI are correlated with angiogenesis and biologic aggressiveness of rectal cancer using other software [14].All included patients underwent CRT within a month after MRI (median 10, range 0**−**25 days) and underwent complete resection of the tumor as follows: lower anterior resection (n = 28), proctosigmoidectomy (n = 4), abdominoperineal resection (n = 3), proctocolectomy (n = 1), and endoscopic resection (n = 1). Radiation therapy of 50.4 Gy was delivered to the pelvis in 36 patients and 45 Gy was delivered in one patient. Twenty-two patients were treated with 5-fluorouracil plus leucovorin and 15 patients with capecitabine.

### 2.2. MR Imaging Techniques

All MRI studies were performed using a 3T MR scanner (Magnetom Verio; Siemens Medical Solutions, Erlangen, Germany) with six-channel phased-array surface coil (Body Matrix) combined with up to six elements of the integrated spine coil. Before MR scanning, approximately 50-100 mL of sonography transmission gel was administered for appropriate distension of the rectum, which assisted in delineating the tumor, particularly in small tumors. The MR images were obtained using the following sequences. First, a sagittal image was obtained with a T2-weighted fast spin-echo sequence. A plane perpendicular to the long axis of the rectal cancer was selected for axial scanning, covering the rectum with the lower edge at least 10 cm below the symphysis pubis and the upper edge below the sacral promontory.

Then, an oblique axial T1-weighted fast spin-echo sequence (TR/TE of 750/10; flip angle of 150°; field of view [FOV] of 200 × 200 mm; matrix size of 320 × 224; 2 NEX; slice thickness of 5 mm with no gap; and acquisition time of 4 minutes 31 seconds) and an oblique axial T2-weighted fast spin-echo sequence (TR/TE of 4000/118; flip angle of 140°; FOV of 200 × 200 mm; spectral width of 363 hz/pixel; matrix size of 320x224; 2 NEX; slice thickness of 5 mm with no gap; acquisition time of 3 minutes 27 seconds) were applied. Diffusion-weighted MR images were acquired on the sagittal and oblique axial planes using the single shot-echo planar imaging technique with b of 0, 500, and 1000 seconds/mm^2^; TR/TE of 6100/83; FOV of 200 mm; matrix size of 104 × 73; 2 NEX; slice thickness of 5 mm with no slice gap; and an acquisition time of 2 minutes 30 seconds. DCE-MRI included two precontrast T1-weighted volumetric interpolated breath-hold examinations (3D VIBE, TR/TE of 5.1/1.8, FOV 250 × 250 mm, matrix 192 × 138, 20 axial slices [slice thickness, 5 mm]) with different flip angles (2°, 15°) to determine the T1 relaxation time in the tissue before the arrival of contrast agent. This imaging was followed by a DCE series with fat suppression on the axial plane with TR/TE of 4.3/1.47; flip angle of 15; slice thickness of 5.0 mm; acquisition time of 4 minute 35 seconds; and an intravenous bolus injection of 0.1 mmol/kg gadobutol (Gadovist, Schering, Berlin, Germany) at a rate of 3 mL/s, followed by a 25 mL saline flush.

### 2.3. Image Analysis

Perfusion parametric maps were obtained using dedicated DCE-MRI software (Olea Sphere; Olea Medical Solutions, La Ciotat, France) with Tofts model implementation [1, 15].

The arterial input function was selected automatically using a cluster analysis algorithm individually.

For voxel-wise histogram analysis of DCE-MRI perfusion parameters, tumor ROIs were manually drawn along the edges of the tumors on T2-weighted axial images section by section at a thickness of 5 mm for the entire tumor, while avoiding areas of necrosis/cystic area or hemorrhage by two abdominal radiologists (S.N.O and M.H.C with 16 and 6 years of experience) independently. ROIs were copied and pasted over automatically driven perfusion maps from the software. Then, the following histogram analysis values of each perfusion parameter were derived: mean; minimum; maximum; standard deviation (SD); mode (the value exhibiting the highest peak on the histogram); skewness; kurtosis; 10th, 20th, 30th, 40th, 50th, 60th, 70th, 80th, and 90th percentiles (the nth percentile is the point at which n% of the voxel values that form the histogram are found to the left) of the DCE-MRI parameters, composed of the volume transfer constant between the blood plasma and EES (K^trans^, min^−1^); the rate constant between EES and the blood plasma (k_ep_, min^−1^); volume of EES space per unit volume of tissue (v_e_); fractional blood-plasma volume (v_p_); and AUC (mM·s). Skewness represents the degree of asymmetry of a distribution. Negative skewness indicates that the distribution is concentrated on the right of the figure, and positive skewness indicates the converse distribution pattern. Kurtosis represents the sharpness of the peaked of the distribution. Higher kurtosis indicates a shaper peak.

**Figure 1:**
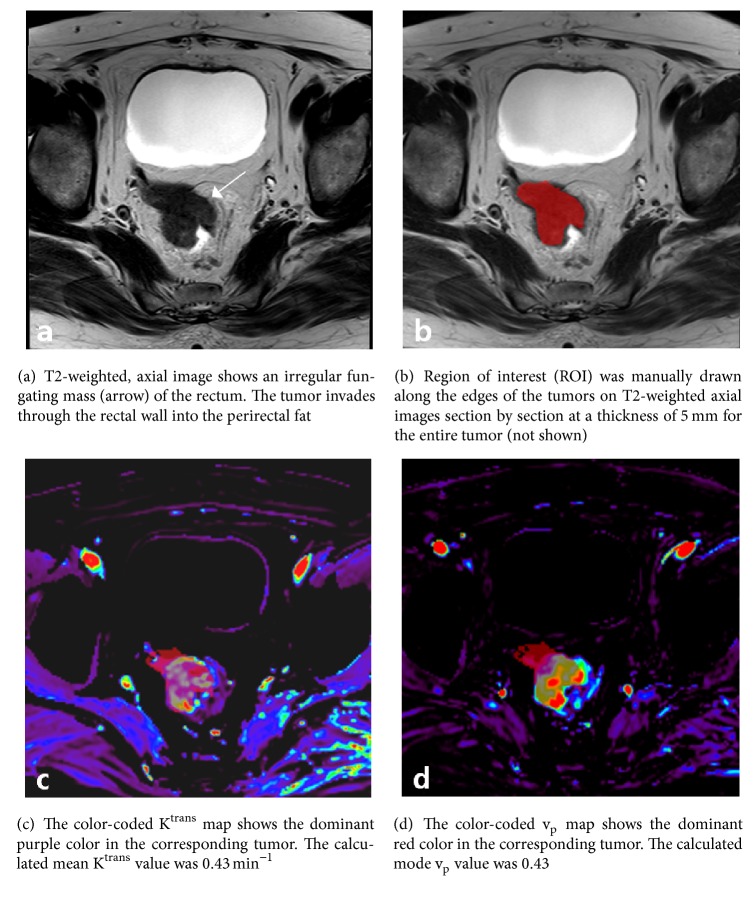
Rectal carcinoma in a 66-year-old female patient with perineural invasion and KRAS gene mutation (+).

**Figure 2:**
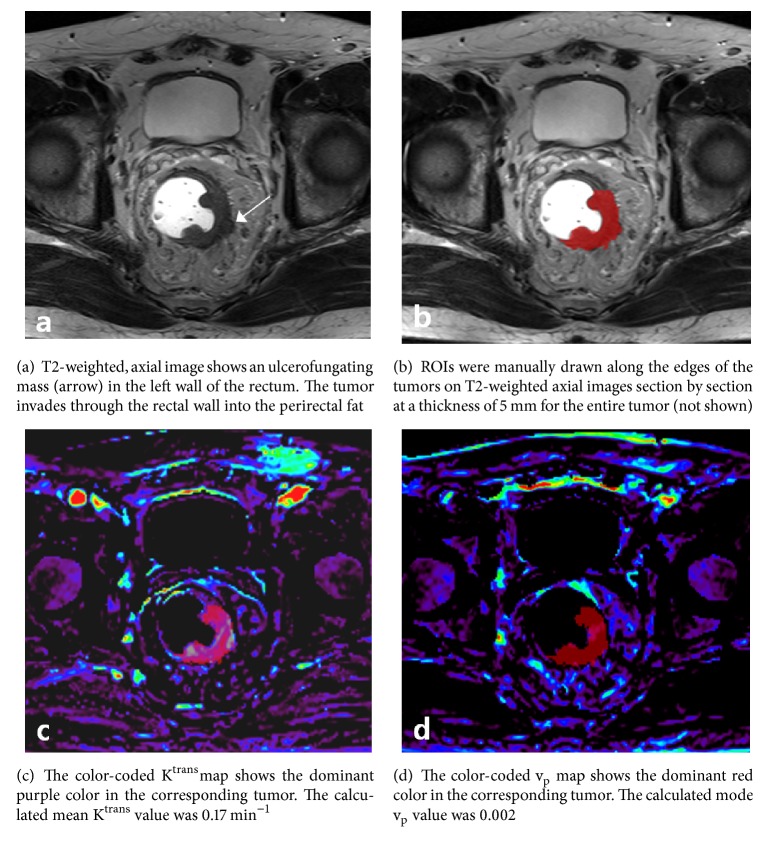
Rectal carcinoma in a 63-year-old male patient without perineural invasion and KRAS gene mutation (-).

Representative cases for histogram analysis of DCE-MRI are shown in Figures 1 and 2.

### 2.4. Histopathologic Analysis

Histopathologic information was obtained from pathology reports. We assessed morphological factors, including depth of invasion (T stage), lymph node metastasis (N stage), and the presence of lymphatic, vascular, and perineural invasion (PNI) as well as biologic markers including expression of EGFR, KRAS gene mutations, and tumor regression grade (TRG) as described by Dworak et al.[16], indicating pathologic grading of regression following neoadjuvant CRT. Tumor regression was classified according to the following five grades: Grade 0, no regression; Grade 1, dominant tumor mass with obvious fibrosis and/or vasculopathy; Grade 2, dominantly fibrotic changes with few tumor cells or groups (easy to find); Grade 3, very few (difficult to find microscopically) tumor cells in fibrotic tissue with or without mucous substance; and Grade 4, no tumor cells, only fibrotic mass (total regression or response).

### 2.5. Statistical Analysis

Statistical analyses were performed using statistical software R version 3.2.1[17] and MedCalc, version 11.5.0.0 [MedCalc, Mariakerke, Belgium]). To assess interobserver reliability of the DCE-MRI parameters, measurements were analyzed using the intraclass correlation coefficient (lower than 0.40, poor agreement; 0.40–0.75, fair to good agreement; and higher than 0.75, excellent agreement). The cases were assigned to groups based on histologic results including depth of invasion (T stage), lymph node metastasis (negative versus positive), lymphovascular invasion (negative versus positive), PNI (negative versus positive), EGFR expression (negative versus positive), and KRAS gene mutation (negative versus positive). To assess neoadjuvant CRT response predictability, the patents were also divided into groups of TRG nonresponders (Grades 0, 1, and 2) and TRG responders (Grades 3 and 4) and complete response (CR) group and non-CR group. The values from histogram analysis of DCE-MRI parameters (K^trans^, k_ep_, v_e_, v_p_, and AUC; mean, minimum, maximum, SD, mode, skewness, kurtosis, 10th, 20th, 30th, 40th, 50th, 60th, 70th, 80th, and 90th percentile value) are compared between the groups using the Mann–Whitney U test with the moonBook package [18].

For the parameters that demonstrated statistically significant difference between the groups, receiver operating characteristics (ROC) curve analysis was performed to calculate the sensitivity, specificity, and diagnostic accuracy.

## 3. Results

### 3.1. Correlation with Prognostic Histologic Results and DCE-MRI Parameters

Histogram analysis measurements of perfusion parameters showed overall excellent interreader agreement except for some minimum or lower percentile measurements.  Table 1 summarizes the interobserver agreement correlation coefficients using the corresponding intraclass correlation coefficients.

Comparisons of DCE-MRI parameters of rectal cancer by group, classified according to histologic results and molecular biology, are summarized in Table 2.

In patients with lymph node metastasis, AUC skewness was significantly lower than that in patients without lymph node metastasis (-0.4; median [-0.7,-0.2; interquartile range] versus -0.2 [-0.3,0.1],* p* = 0.016).Therefore, a larger proportion of higher AUC values were present in the nodal metastasis group compared to the group with nonnodal metastasis. The area under the ROC curve (*A*_z_) of AUC skewness was 0.744 (95% CI: 0.565-0.922; sensitivity 69.2%, specificity 79.2%) for reader 1 and 0.753 (95% CI: 0.583-0.923; sensitivity 69.2%, specificity 75.0%) for reader 2. AUC kurtosis and v_p_ kurtosis also showed higher values in the nodal metastasis group, which was represented by a sharper histogram peak, in reader 1 only.

The v_p_-associated histogram values (mean, 10th−80th percentile, skewness, kurtosis, and mode) showed statistically significant correlation with PNI. ROC curve analyses revealed that mode v_p_ showed the best diagnostic performance of PNI (*A*_z_ of mode v_p_0.859; 95% CI: 0.698-1; sensitivity 87.5%, specificity 81.5% for reader 1; *A*_z_ of modev_p_0.783; 95% CI: 0.591-0.976; sensitivity 62.5%, specificity 89.3% for reader 2).

The K^trans^ (mean, SD, 50th−90th percentile, and mode) and k_ep_ histogram values (mean, 30th−90th percentile, and kurtosis) were significantly higher in the group with KRAS gene mutation and v_e_ kurtosis was lower in KRAS-mutated than in nonmutated tumors. ROC curve analyses showed that mean K^trans^ and mode K^trans^ demonstrated excellent diagnostic performance of KRAS gene mutation (*A*_z_ of mean K^trans^ 0.788, 95% CI: 0.610-0.967; sensitivity 76.9%, specificity 81.2%; *A*_z_ of mode K^trans^0.793, 95% CI: 0.624-0.963; sensitivity 100%, specificity 56.2% for reader 1).

Other histologic (T stage, lymphatic invasion, and vascular invasion) and immunohistochemical (EGFR expression) results were not associated with any difference in DCE-MRI parameters.

### 3.2. Correlation with Treatment Response after Neoadjuvant CRT and DCE-MRI Parameters

Of the total 37 patients, 10 were in TRG 1, 19 were in TRG 2, 1 was in TRG 3, and 7 were in TRG 4 (CR). The mean K^trans^ values of the responder and nonresponders groups were similar (0.4; median [0.3, 0.5; interquartile range] versus 0.4[0.3, 0.5],* p* = 0.685). The mean k_ep_was lower in the TRG responder group compared to the TRG nonresponder group, but the difference was not statistically significant (1.0 ± 0.5 versus 1.0 ± 0.3,* p* = 0.760).

The mean K^trans^ and mean k_ep_ were lower in the CR group compared to the non-CR group (0.3[0.3; 0.4] versus 0.4[0.3; 0.5],* p* = 0.461; 1.0 [0.9,1.0] versus 1.2 [0.8; 1.4],* p* = 0.332, respectively), but the differences were not statistically significant. No other DCE-MRI parameter histogram analysis values were significantly correlated with CRT treatment response. The mean, maximum, skewness, and kurtosis of K^trans^ and k_ep_, based on TRG and CR, are summarized in Table 3.

## 4. Discussion

The aim of the present study was to explore the role of histogram analysis of model-based perfusion parameters from DCE-MRI in rectal cancer for discriminating tumor characteristics and predicting CRT response. Our results showed that histogram values from DCE-MRI parameters correlated with prognostic factors including LN metastasis, PNI, and KRAS gene mutation. The histogram analysis values of DCE-MRI parameters were not correlated with pathologic CRT response.

Previous studies have reported discrepant results regarding the correlation of TNM staging and DCE-MRI parameters. Yao et al. suggested that K^trans^ positively correlates with LN metastasis [5]. However, Kim et al. reported no relationship between TN staging and K^trans^ and v_e_ [6]. In our study, K^trans^, k_ep_, and v_e_ revealed no correlation with TNM staging, and the AUC data of the group with nodal metastasis demonstrated wider spread to the right of the mean compared to that of the group with nonnodal metastasis, illustrating that a larger proportion of patients with nodal metastasis had higher AUC values than patients without nodal metastasis.

To the best of our knowledge, there have been no studies regarding the correlations between the PNI of rectal cancer and DCE-MRI parameters. Our present study showed a significant correlation between PNI and v_p_. The presence of PNI in rectal cancer is associated with a significantly worse prognosis [19, 20], indicating that a high v_p_ is a poor prognostic factor.

In patients with metastatic colorectal cancer, treatment using EGFR-directed antibodies such as cetuximab or panitumumab is recommended. However, KRAS (exon2 or nonexon2) or NRAS mutations are known to be resistant to EGFR-targeting agents; therefore, anti-EGFR therapy cannot be used in patients with RAS gene mutations. In the present study, there were no patients with NRAS mutation, and 13 patients (44. 8%, 13/29) with KRAS mutation. Most histogram values of K^trans^ and k_ep_ were higher in the KRAS mutation group. In our previous study, there was also a higher mean K^trans^ in the group with KRAS mutation, although the difference did not reach statistical significance[14]. However, the present study showed statistical significance of higher K^trans^ and k_ep_ correlating with presence of aKRAS gene mutation. It is well known that the mutant KRAS oncogene can induce or strongly upregulate various proangiogenic factors such as vascular endothelial growth factor/vascular permeability factor (VEGF/VPF) and transforming growth factors *β* (TGF- *β*) or *α* (TGF- *α*) in a cascade manner. Although the precise mechanism has not been discovered, the current study suggests the possibility of MRI-derived perfusion parameters reflecting an event at the genetic level of tumorigenesis[21, 22].Although further studies of clinical validity with a larger sample size are required, K^trans^ or k_ep_ may be important imaging biomarkers in predicting an individual's response to anti-EGFR therapy, even before genotyping.

Contrary to the significant results regarding the usefulness of mean K^trans^ for response assessment or prediction of CRT in previous studies[2–4, 23], our study demonstrated no correlation of histogram values of K^trans^, k_ep_, or v_e_ with CRT response. However, several studies have reported similar results. Lim et al.[2]demonstrated that K^trans^ was not predictive of TRG, and Kim et al. [3] also reported that K^trans^, k_ep_, and v_e_ are not useful to assess or predict CR. Furthermore, Intven et al. [23] revealed that changes in K^trans^ after CRT have no additive value for response assessment in the combination study of T2-weighted MR volumetry, diffusion-weighted MR imaging, and DCE-MRI. Further studies with larger sample sizes are needed to investigate clinical validation and additive values of perfusion MRI for response assessment or prediction of CRT.

Our study has several limitations. First, this is a retrospective study and therefore has an unavoidable selection bias. Second, the sample size was relatively small and was thus insufficient to suggest optimal threshold values of DCE-MRI parameters for predicting prognosis. Third, we did not analyze the MRI after CRT and thus cannot assess the changes in perfusion parameters after CRT. However, in a clinical setting, there is actually less interest in assessing treatment response after CRT compared to predicting response before CRT. For this reason, we performed this study to explore the role of DCE-MRI in predicting treatment response before CRT. These preliminary results suggest that a larger proportion of higher AUC skewness was present in LN metastasis group and a higher v_p_ histogram value was present in rectal cancer with PNI. In addition, K^trans^ and k_ep_ histogram parameters showed difference according to the KRAS mutation, demonstrating the utility of the histogram of perfusion parameters derived from DCE-MRI as potential imaging biomarkers of tumor characteristics and genetic features.

## Figures and Tables

**Table 1:** Interobserver intraclass correlation coefficient for measurements of perfusion parameters.

Parameter	K^trans^	v_p_	AUC	k_ep_	v_e_
Mean	0.971 (0.943, 0.985)	0.995 (0.990, 0.997)	0.982 (0.965, 0.991)	0.993 (0.987, 0.997)	0.996 (0.993, 0.998)
Minimum	0.435 (0.129, 0.665)	0.414 (0.104, 0.651)	0.665 (0.435, 0.814)	0.747 (0.559, 0.863)	0.889 (0.794, 0.942)
10th percentile	0.264 (-0.065, 0.542)	0.973 (0.948, 0.986)	0.907 (0.826, 0.952)	0.977 (0.955, 0.988)	0.998 (0.996, 0.999)
20th percentile	0.254 (-0.077, 0.534)	0.985 (0.970, 0.992)	0.925 (0.859, 0.961)	0.967 (0.936, 0.983)	0.998 (0.995, 0.999)
30th percentile	0.413 (0.103, 0.650)	0.989 (0.979, 0.995)	0.936 (0.879, 0.967)	0.979 (0.960, 0.989)	0.998 (0.997, 0.999)
40th percentile	0.788 (0.623,0.886)	0.993 (0.986, 0.996)	0.970 (0.941, 0.984)	0.987 (0.974, 0.993)	0.997 (0.995, 0.999)
50th percentile	0.928 (0.863, 0.963)	0.995 (0.990, 0.997)	0.981 (0.963, 0.991)	0.987 (0.974, 0.993)	0.998 (0.996, 0.999)
60th percentile	0.993 (0.986, 0.996)	0.997 (0.994, 0.998)	0.982 (0.964, 0.991)	0.990 (0.981, 0.995)	0.994 (0.988, 0.997)
70th percentile	0.996 (0.993, 0.998)	0.996 (0.993, 0.998)	0.991 (0.982, 0.995)	0.991 (0.982, 0.995)	0.991 (0.982, 0.995)
80th percentile	0.996 (0.993, 0.998)	0.994 (0.988, 0.997)	0.995 (0.989, 0.997)	0.993 (0.986, 0.996)	0.977 (0.955, 0.988)
90th percentile	0.996 (0.991, 0.998)	0.995 (0.991, 0.998)	0.996 (0.992, 0.998)	0.994 (0.988, 0.997)	0.994 (0.988, 0.997)
Maximum	0.970 (0.941, 0.984)	0.972 (0.947, 0.986)	1.000	0.997 (0.994, 0.999)	1.000
Standard deviation	0.981 (0.965, 0.989)	0.993 (0.987, 0.996)	0.962 (0.931, 0.979)	0.996 (0.992, 0.998)	0.961 (0.930, 0.979)
Mode	0.965 (0.933, 0.982)	0.925 (0.856, 0.962)	0.957 (0.912, 0.978)	0.835 (0.700, 0.912)	0.998 (0.996, 0.999)
Skewness	0.955 (0.913, 0.977)	0.969 (0.940, 0.984)	0.950 (0.904, 0.974)	0.990 (0.980, 0.995)	0.988 (0.978, 0.994)
Kurtosis	0.888 (0.792, 0.941)	0.935 (0.877, 0.966)	0.920 (0.849, 0.958)	0.984 (0.968, 0.992)	0.967 (0.936, 0.983)

*Note.* Data in parentheses are 95% confidence intervals.

**Table 2:** Correlation of histogram analysis of perfusion parameters with biologic aggressiveness.

	Parameter	Reader 1				Reader 2			
		Yes (n = 24)	No (n = 13)	*P *value^*∗*^	A_z_^†^	Yes (n = 24)	No (n = 13)	*P *value^*∗*^	A_z_^†^
Biologic aggressiveness	AUC skewness	-0.4 (-0.7;-0.2)	-0.2 (-0.3;0.1)	**0.016**	0.744	-0.4(-0.6;-0.2)	-0.2(-0.3;0.2)	**0.012**	0.753
	AUC kurtosis	-0.1 (-0.5;0.1)	-0.6 (-0.9;-0.1)	**0.036**	0.712	-0.4 (-0.6;0.2)	-0.6(-0.8;-0.1)	**0.098**	0.667
	v_p_ kurtosis	-0.2 (-0.6;0.4)	0.4 (-0.1;1.6)	**0.036**	0.712	-0.3 (-0.5;0.2)	0.5 (-0.2;1.7)	**0.052**	0.696

		Yes (n = 8)	No (n = 29)	*P *value	A_z_^*∗*^	Yes (n = 8)	No (n = 29)	*P *value	A_z_^*∗*^

PNI	v_p_ mean	0.3 (0.2;0.3)	0.1 (0.1;0.2)	**0.042**	0.737	0.3 (0.2;0.3)	0.1 (0.1;0.2)	**0.046**	0.733
	v_p_ 10th percentile	0.1 (0.1;0.1)	0.0 (0.0;0.0)	**0.011**	0.797	0.1 (0.1;0.1)	0.0 (0.0;0.0)	**0.013**	0.789
	v_p_ 20th percentile	0.1 (0.1;0.2)	0.1 (0.0;0.1)	**0.022**	0.767	0.2 (0.1;0.2)	0.1 (0.0;0.1)	**0.035**	0.746
	v_p_ 30th percentile	0.2 (0.1;0.2)	0.1 (0.0;0.1)	**0.024**	0.763	0.2 (0.1;0.2)	0.1 (0.1;0.1)	**0.039**	0.741
	v_p_ 40th percentile	0.2 (0.2;0.3)	0.1 (0.0;0.1)	**0.027**	0.759	0.2 (0.1;0.3)	0.1 (0.1;0.1)	**0.042**	0.737
	v_p_ 50th percentile	0.3 (0.2;0.3)	0.1 (0.1;0.2)	**0.029**	0.754	0.3 (0.2;0.3)	0.1 (0.1;0.2)	**0.035**	0.746
	v_p_ 60th percentile	0.3 (0.2;0.4)	0.2 (0.1;0.2)	**0.032**	0.750	0.3 (0.2;0.4)	0.1 (0.1;0.2)	**0.035**	0.746
	v_p_ 70th percentile	0.4 (0.2;0.4)	0.2 (0.1;0.2)	**0.035**	0.746	0.4 (0.2;0.4)	0.2 (0.1;0.2)	**0.039**	0.741
	v_p_ 80th percentile	0.4 (0.3;0.5)	0.2 (0.2;0.3)	**0.046**	0.733	0.4 (0.3;0.5)	0.2 (0.2;0.3)	**0.042**	0.746
	v_p_ skewness	0.4 (0.1;0.5)	0.8 (0.6;1.3)	**0.022**	0.767	0.3 (0.2;0.6)	0.8 (0.6;1.3)	**0.020**	0.772
	v_p_ kurtosis	-0.4 (-0.7;-0.1)	0.4 (-0.2;1.4)	**0.018**	0.776	-0.4 (-0.7;-0.1)	0.4 (-0.3;1.6)	**0.035**	0.746
	**v**_**p**_ ** mode**	0.2 (0.1;0.4)	0.0 (0.0;0.1)	**0.002**	**0.859**	0.1 (0.1;0.3)	0.0 (0.0;0.1)	**0.016**	**0.783**

		Yes (n = 13)	No (n = 16)	*P *value	A_z_^*∗*^	Yes (n = 13)	No (n = 16)	*P *value	A_z_^*∗*^

KRAS mutation	**K** ^**trans**^ **mean**	0.5 (0.4;0.5)	0.3 (0.2;0.4)	**0.009**	**0.788**	0.5 (0.4;0.5)	0.3 (0.2;0.4)	**0.010**	**0.784**
	K^trans^SD	0.3 (0.3;0.5)	0.2 (0.1;0.3)	**0.020**	0.755	0.3 (0.3;0.5)	0.2 (0.1;0.4)	**0.035**	0.731
	K^trans^50th percentile	0.4 (0.2;0.5)	0.2 (0.2,0.3)	**0.039**	0.726	0.4 (0.2;0.5)	0.2 (0.2,0.3)	**0.048**	0.716
	K^trans^60th percentile	0.5 (0.3;0.6)	0.3 (0.2;0.4)	**0.035**	0.731	0.5 (0.4;0.5)	0.3 (0.2;0.4)	**0.048**	0.716
	K^trans^70th percentile	0.6 (0.4;0.7)	0.4 (0.3;0.5)	**0.028**	0.740	0.6 (0.4;0.7)	0.4 (0.3;0.5)	**0.032**	0.736
	K^trans^80th percentile	0.8 (0.5;0.9)	0.5 (0.3;0.6)	**0.014**	0.769	0.8 (0.5;0.9)	0.5 (0.3;0.6)	**0.023**	0.750
	K^trans^90h percentile	0.8 (0.8;1.4)	0.6 (0.4;0.9)	**0.023**	0.750	0.9 (0.8;1.3)	0.6 (0.4;0.9)	**0.039**	0.726
	**K** ^**trans**^ **mode**	1.3 (0.8;1.8)	0.6 (0.0;1.1)	**0.007**	**0.793**	1.3 (0.8;1.8)	0.6 (0.1;1.1)	**0.007**	**0.793**
	k_ep_ mean	1.4 (1.2; 1.5)	0.9 (0.3;1.3)	**0.018**	0.760	1.3 (1.2; 1.5)	1.0 (0.3;1.3)	**0.044**	0.721
	k_ep_30th percentile	0.7 (0.5;0.8)	0.5 (0.2;0.7)	**0.025**	0.745	0.7 (0.6;0.8)	0.5 (0.2;0.6)	**0.032**	0.736
	k_ep_40th percentile	0.9 (0.8;1.1)	0.6 (0.2;0.8)	**0.028**	0.740	0.9 (0.8;1.1)	0.7 (0.2;0.8)	**0.028**	0.740
	k_ep_50th percentile	1.1 (0.9;1.4)	0.8 (0.2;1.0)	**0.018**	0.760	1.1 (1.0;1.2)	0.8 (0.2;1.0)	**0.039**	0.726
	k_ep_60th percentile	1.4 (1.1;1.6)	0.9 (0.3;1.2)	**0.020**	0.755	1.4 (1.2;1.5)	1.0 (0.3;1.3)	**0.035**	0.731
	k_ep_70th percentile	1.8 (1.4;1.8)	1.1 (0.3; 1.5)	**0.028**	0.740	1.7 (1.4;1.9)	1.2 (0.4;1.5)	**0.032**	0.736
	k_ep_80th percentile	2.1 (1.8;2.5)	1.4 (0.4;1.9)	**0.016**	0.764	2.1 (1.7;2.7)	1.5 (0.4;2.0)	**0.032**	0.736
	k_ep_90th percentile	2.7 (2.4;3.1)	1.9 (0.6;2.7)	**0.025**	0.745	2.7 (2.3;3.1)	1.9 (0.6;2.8)	**0.048**	0.716
	v_e_ kurtosis	0.5 (-0.6;1.9)	1.3 (0.9;5.1)	**0.035**	0.731	0.4 (-0.6;1.4)	2.0 (0.7;5.6)	**0.018**	0.760

*Note.* All figures of perfusion parameters in the above table have been rounded to one decimal place and are presented as median value (interquartile range) according to the data distribution.

Numbers in bold are statistically significant *P* -values. Parameters in bold are high in area under the ROC curve.

AUC, area under the concentration curve; PNI, perineural invasion; SD, standard deviation.

^*∗*^Determined with the Mann-Whitney U test.

^†^ *Az*= area under the ROC curve.

**Table 3:** Correlation with treatment response of neoadjuvant chemoradiotherapy after rectal cancer.

Treatment Response	Parameter	Reader 1			Reader 2		
		TRG0,1,2(n=29)	TRG 3,4 (n=8)	*P* value^*∗*^	TRG0,1,2(n=29)	TRG3,4 (n=8)	*P* value^*∗*^

**TRG**	K^trans^mean	0.4 (0.3;0.5)	0.4 (0.3;0.5)	0.685	0.4 (0.3;0.5)	0.4 (0.3;0.4)	0.854
	K^trans^maximum	1.3 (0.7;1.8)	0.9 (0.7;1.5)	0.685	1.3 (0.7;1.8)	0.9 (0.7;1.5)	0.605
	K^trans^skewness	0.9 (0.2;1.6)	0.8 (0.1;1.5)	0.912	0.8 (0.3;1.6)	0.8 (0.1;1.6)	0.912
	K^trans^kurtosis	0.4 (-0.9;2.1)	-0.1 (-1.2;1.9)	0.507	0.2 (-0.9;2.7)	-0.2 (-1.1;2.5)	0.825
	k_ep_mean	1.2 (0.8;1.4)	1.0 (0.9;1.1)	0.685	1.1 (0.7;1.3)	1.0(0.9;1.1)	0.483
	k_ep_maximum	3.3 (2.7;4.0)	3.5 (2.7;4.2)	1.000	3.5 (2.7;4.0)	3.5 (2.7;4.2)	0.971
	k_ep_skewness	1.3 (0.9;1.7)	1.3 (0.9;1.8)	0.971	1.3 (0.9;1.7)	1.4 (0.9;1.8)	0.941
	k_ep_kurtosis	1.1 (0.2;4.1)	2.1 (-0.1;3.9)	0.941	1.3 (0.3;3.6)	2.2 (-0.1;4.0)	0.941

		CR (n=7)	nonCR (n=30)	*P* value	CR (n=7)	nonCR (n=30)	*P* value

**CR**	K^trans^mean	0.3 (0.3;0.4)	0.4 (0.3;0.5)	0.461	0.4 (0.3;0.4)	0.4 (0.3;0.5)	0.587
	K^trans^maximum	1.0 (0.7;1.5)	1.2 (0.7;1.8)	0.816	1.0 (0.7;1.5)	1.2 (0.7;1.8)	0.727
	K^trans^skewness	1.0 (0.4;1.5)	0.8 (0.1; 1.6)	0.670	1.0 (0.4;1.6)	0.8 (0.1;1.6)	0.614
	K^trans^kurtosis	0.3 (-0.8;1.9)	0.3(-0.9;2.1)	0.907	0.1 (-0.8;2.5)	0.1(-0.9;2.7)	0.786
	k_ep_mean	1.0 (0.9;1.0)	1.2 (0.8;1.4)	0.332	0.9 (0.9;1.0)	1.2 (0.7;1.4)	0.201
	k_ep_maximum	3.8 (2.9; 4.2)	3.3 (2.7;4.0)	0.756	3.8 (2.9;4.2)	3.4 (2.7;4.0)	0.786
	k_ep_skewness	1.5 (1.1;1.8)	1.3 (0.7;1.7)	0.510	1.6 (1.2;1.8)	1.3 (0.8;1.7)	0.438
	k_ep_kurtosis	3.1 (0.8;3.9)	1.1(-0.5;4.1)	0.535	3.1 (0.9;4.0)	1.3(-0.5;3.6)	0.535

*Note.* TRG, tumor regression grade; TRG0, no regression; TRG1, dominant tumor mass with obvious fibrosis and/or vasculopathy; TRG2, dominantly fibrotic changes with few tumor cells or groups; TRG3, very few tumor cells in fibrotic tissue with or without mucous substance; TRG4, no tumor cells, only fibrotic mass; TRG nonresponders (Grades 0,1, and 2) and TRG responders (Grades 3 and 4); CR, complete response.

All figures of perfusion parameters in the above table have been rounded to one decimal place and are presented as median value (interquartile range) according to the data distribution.

^*∗*^Determined with the Mann-Whitney U test.

## Data Availability

This study is based on medical images of patients. Sharing data is believed to be a possible source of legal and ethical issues. The software used for image analysis is currently commercially available.
